# Association of *P2Y12* Polymorphisms With the Risk of Ischemic Stroke Subtypes

**DOI:** 10.31083/RN45447

**Published:** 2026-02-06

**Authors:** Conglian Wu, Yabin Chen, Jintu Chen, Xiaolan Wei, Zhishan Zhang

**Affiliations:** ^1^Department of Clinical Laboratory, Quanzhou First Hospital Affiliated to Fujian Medical University, 362000 Quanzhou, Fujian, China; ^2^Department of Neurology, Quanzhou First Hospital Affiliated to Fujian Medical University, 362000 Quanzhou, Fujian, China

**Keywords:** ischemic stroke, receptors, purinergic P2Y12, polymorphism, genetic, large-artery atherosclerosis, nomograms

## Abstract

**Background:**

To evaluate the association of the purinergic receptor P2Y, G-protein coupled, 12 (*P2Y12*) gene polymorphisms with susceptibility to different etiological stroke subtypes.

**Methods:**

A total of 459 first-ever acute ischemic stroke patients were classified into large-artery atherosclerosis (LAA, n = 163), small-vessel occlusion (SVO, n = 204), and cardioembolism (CE, n = 92) based on the Trial of Org 10172 in Acute Stroke Treatment (TOAST) criteria. Direct sequencing was used to screen these three stroke subtypes and non-stroke controls for *P2Y12* polymorphisms: a T→C transition at 744 nucleotides (nt) downstream of intron 5's start site (*i-T744C*) and a C→T transition at 34 nt downstream of exon 2's start site (*C34T*). Based on the results of multivariate logistic analyses, a prediction model was established via a nomogram that incorporated genomic and clinical variables to quantify the risk of LAA stroke.

**Results:**

Significant differences in the *P2Y12* *i-T744C* genotype and allele frequencies were observed between LAA patients and controls. After adjusting for confounding factors, the dominant model (*p* = 0.009) and additive model (*p* = 0.023) revealed that the *i-T744C* polymorphism was significantly associated with increased susceptibility to LAA. No significant associations were found for the SVO and CE stroke subtypes. Moreover, the *C34T* polymorphism was not an independent factor for any stroke subtype. We further constructed a nomogram prediction model for LAA stroke based on genomic and clinical variables, including age, hypertension, smoking, high-density lipoprotein cholesterol, and the *i-T744C* polymorphism. This nomogram exhibited satisfactory accuracy and predictive power for LAA stroke, as demonstrated by the area under the curve, calibration plot, and decision curve analysis.

**Conclusion:**

The *P2Y12* *i-T744C* polymorphism may serve as a predictor for LAA stroke. Furthermore, we constructed a genomic-clinical nomogram that may be valuable for predicting LAA stroke risk in the study population.

## 1. Introduction 

The global burden of ischemic stroke (IS) keeps rising [1], with forecasts suggesting that IS-related deaths will increase to approximately 4.9 million worldwide by 2030 [2, 3]. IS is a complex disorder that can be classified into five etiological subtypes according to the Trial of Org 10172 in Acute Stroke Treatment (TOAST) classification: large-artery atherosclerosis (LAA), small-vessel occlusion (SVO), cardioembolism (CE), undetermined, and other determined etiology [4]. The clinical manifestations, targeted therapeutic strategies, and prognostic outcomes across the different etiological subtypes are distinct [4, 5], underscoring the significance of subtype-specific research in IS. The primary cause of LAA stroke is atherosclerotic plaque formation and subsequent rupture of large cerebral arteries [6]. LAA typically manifests with severe focal neurological deficits (e.g., hemiplegia, aphasia), accompanied by a high susceptibility to early neurological deterioration [4].

The P2Y purinoceptor 12 (P2Y12 receptor) gene (herein referred to as the *P2Y12* gene) localizes to chromosome 3, long arm bands q21 to q25 (*3q21-q25*) and encodes a 342-residue, G-protein-coupled receptor on the surface of platelets, microglia, and vascular smooth muscle cells (VSMCs) [7]. The adenosine diphosphate (ADP)-mediated P2Y12 receptor signaling pathway can promote platelet activation and aggregation, pro-inflammatory cytokine release, formation of platelet-leukocyte aggregates, and VSMC migration and proliferation [8]. These processes contribute to the progression of atherosclerosis and thrombosis. Consistent with the essential role of the P2Y12 receptor in atherosclerosis and pathological thrombosis, one of its single-nucleotide polymorphisms (SNPs), *i-T744C* (rs2046934, a T→C transition at 744 nucleotides (nt) downstream of intron 5’s start site), was observed to significantly influence ADP-induced platelet aggregation [9, 10, 11]. *i-T744C* was also associated with increased susceptibility to atherosclerosis and related complications, such as coronary artery disease (CAD) and myocardial infarction [12, 13]. Prior studies linking *P2Y12* polymorphisms to IS susceptibility yielded inconsistent results [14, 15]. However, these studies did not stratify IS into subtypes, thus potentially overlooking a possible subtype-specific effect of *P2Y12* polymorphisms. Investigation of the association of *P2Y12* polymorphisms with specific IS subtypes can overcome the potential confounding effect of subtype heterogeneity, thereby improving the accuracy of genetic risk stratification. As far as we are aware, no such studies have yet been conducted.

Nomograms are a clinically valuable instrument for integrating diverse data types and can be used for multi-disease risk assessment with increased precision. The LAA subtype is a multi-factorial disease attributable to an interplay of multiple factors [16]. Integration of these variables into a predictive model may enhance the diagnostic accuracy of LAA. These considerations motivated us to develop a nomogram that integrates well-established genetic and clinical variables, enabling quantification of the weighted contribution of each input variable and making it intuitive for clinical practice.

Therefore, the aim of this study was to elucidate the relationship between two common *P2Y12* polymorphisms [*i-T744C* and *C34T* (rs6785930, a C→T transition at 34 nt downstream of exon 2’s start site)] and the susceptibility to three major IS subtypes (LAA, SVO, and CE), as defined by TOAST criteria. We also developed a quantitative genetic-clinical nomogram, with the aim of improving the ability to predict LAA risk.

## 2. Materials and Methods

### 2.1 Study Population

Patients admitted to Quanzhou First Hospital in Fujian Province, China, for their first-ever IS between February 2024 and March 2025 were recruited to this research project. The diagnosis of IS was based on World Health Organization (WHO) criteria and was radiologically confirmed by computed tomography scans (CT) or magnetic resonance imaging (MRI) [2]. Eligible patients were categorized into five etiological subtypes by experienced neurologists and according to TOAST criteria [4, 17]. The diagnostic criteria for LAA, SVO and CE stroke were: (1) LAA, cerebral hypodensities with a diameter of >15 mm on CT or MRI, and stenosis >50% in the appropriate extracranial or intracranial arteries confirmed by duplex imaging or arteriography, with exclusion of cardiac embolism; (2) SVO, clinical manifestations compatible with lacunar syndromes, accompanied by brain stem or subcortical lesions with a diameter <15 mm on CT or MRI, with exclusion of cardiac embolism and >50% large artery stenosis; (3) CE, the presence of one of high-risk or medium-risk sources of cardiac embolism, with exclusion of embolic sources of large artery atherosclerotic origin. Transthoracic echocardiography (TTE) and/or transesophageal echocardiography (TEE) were performed to exclude cardioembolic sources in the LAA and SVO groups. Inclusion criteria were: (1) first-ever IS and admitted within 1 week of symptom onset; (2) age ≥18 years; (3) established IS etiology (LAA, SVO, or CE); (4) complete data on all variables. Exclusion criteria were: (1) patients with systemic tumors, systemic infection, mental disorders, hematologic disorders, coagulopathic disorders, autoimmune disorders, serious hepatic or renal disorders, or undetermined or unclassified etiologies; (2) incomplete clinical information; (3) prior history of cerebrovascular diseases based on medical records. All patients received guideline-concordant standard care, as recommended by the American Heart Association (AHA)/American Stroke Association (ASA), with no experimental treatments administered during the study period [18].

During the same study period, non-stroke controls (age ≥18 years) who underwent comprehensive neurological and physical examinations were recruited from our hospital’s neurology outpatient clinic via a simple random sampling method. Inclusion criteria for the controls were: no clinical evidence of cerebrovascular diseases; normal cranial CT and/or MRI findings. The exclusion criteria were consistent with those formulated for the case group.

### 2.2 Variables Pool

Data on the genetic variable (*P2Y12 i-T744C* polymorphism) were collected, as well as 12 clinical variables with <10% missing values: age (years), sex (male/female), cigarette smoking (yes/no), alcohol intake (yes/no), hypertension (yes/no), diabetes mellitus (yes/no), ischemic heart disease (yes/no), atrial fibrillation (yes/no), high-density lipoprotein cholesterol (HDL-C, mmol/L), triglyceride (TG, mmol/L), low-density lipoprotein cholesterol (LDL-C, mmol/L), and total cholesterol (TC, mmol/L).

### 2.3 DNA Extraction and Genotyping

Venous whole blood was drawn from participants within 1 week of the IS event and placed into EDTA-K2 anticoagulant tubes. Genomic DNA was extracted from these blood samples using the TIANamp Genomic DNA Kit (DP319-02, TianGen Biotech Co., Beijing, China) and stored at –20 ℃ for subsequent genetic analysis. PCR was performed as follows: denaturation initiation at 94 °C for 5 min, with 30 amplification cycles (94 °C/30 sec, 56 °C/30 sec, and 72 °C/35 sec), a final 10-min extension at 72 °C, and hold at 4 °C [13]. A 50 µL PCR reaction system was utilized, consisting of 25 µL 2X SanTap PCR Mix (B532061, Sangon Biotech Co., Shanghai, China), 3 µL genomic DNA, 4 µL primer sets, and 18 µL sterile water. Genotyping of the amplicons with Sanger sequencing was performed by Sangon Biotech Co. using primers described previously [13].

### 2.4 Statistical Analysis 

Statistical analyses were conducted with IBM SPSS Statistics 27.0 (IBM Corp., Armonk, NY, USA). The nomogram was generated on the Beckman Coulter DxAI platform based on R version 4.2.3 (https://www.xsmartanalysis.com/beckman/login/). Continuous data are expressed as the mean ± SEM, and categorical data as numbers and frequencies. Intergroup differences in variables were analyzed using Student’s *t*-test (continuous data) or χ^2^/Fisher’s exact test (categorical data). The two SNPs in the *P2Y12* gene were assessed for conformance with the Hardy–Weinberg equilibrium using the χ^2^ test, followed by univariate and multivariate logistic regression analyses to evaluate their associations with different IS subtypes.

Non-stroke controls and LAA patients were randomly categorized at a 4:1 ratio. The former (n = 282) served as the training cohort for construction of the model, while the latter (n = 71) served as the validation cohort to assess the model’s robustness. Detailed procedures for construction of the nomogram were as follows: candidate variables associated with LAA stroke (*p* < 0.05) were initially screened from the variable pool in the training cohort using univariate analysis; a multicollinearity test using the Variance Inflation Factor (VIF) was performed for all candidate variables, and those with a VIF >5 were removed from further analyses; the remaining variables in the training cohort were included in multivariable logistic regression to identify independent predictors, which were ultimately used to construct the nomogram for predicting LAA stroke risk. This predictive nomogram was further validated in the internal cohort, using the same predictors as those employed in the training cohort. Net reclassification improvement (NRI) and integrated discrimination improvement (IDI) metrics were applied to quantify the incremental predictive value gained by incorporating the *P2Y12 i-T744C* polymorphism into the LAA stroke risk prediction nomogram. The nomogram’s predictive performance was assessed via the receiver operating characteristic curve (ROC), with calculation of the area under the ROC curve (AUC) to quantify this performance. Concordance between the predicted probabilities and the actual observed results was evaluated via a calibration curve and the Hosmer-Lemeshow test. The net benefit of the predictive model was evaluated by decision curve analysis (DCA). A *p*-value < 0.05 was considered statistically significant.

## 3. Results 

### 3.1 Participant Characteristics 

A total of 459 IS patients with defined etiology participated in this study, comprising 163 (35.5%) patients with LAA stroke, 204 (44.4%) with SVO stroke, and 92 (20.1%) with CE stroke. Demographic and clinical characteristics of the controls and different IS subtypes are presented in Table 1. The average age, hypertension frequency, and HDL-C levels were significantly different between all IS subtypes and controls (all *p* < 0.05). Diabetes mellitus, smoking, and TG levels were associated with LAA and SVO subtypes (all *p* < 0.05), but not with the CE subtype. Additionally, LDL-C levels, ischemic heart disease, and atrial fibrillation were associated with the CE subtype (all *p* < 0.05), but not with LAA and SVO subtypes.

**Table 1.** **Participant characteristics**.

Variable	Controls (n = 190)	LAA stroke (n = 163)	*p*-value	SVO stroke (n = 204)	*p*-value	CE stroke (n = 92)	*p*-value
Age (years)	59.69 ± 9.43	63.12 ± 10.71	0.002*	62.63 ± 10.13	0.004*	68.73 ± 14.11	<0.001*
Men, n (%)	91 (47.9)	92 (56.4)	0.109	95 (46.6)	0.792	56 (60.9)	0.041
Hypertension, n (%)	66 (34.7)	119 (73.0)	<0.001*	144 (70.6)	<0.001*	67 (72.8)	<0.001*
Diabetes mellitus, n (%)	44 (23.2)	73 (44.8)	<0.001*	78 (38.2)	0.001*	27 (29.3)	0.262
Cigarette smoking, n (%)	28 (14.7)	49 (30.0)	<0.001*	59 (28.9)	<0.001*	22 (23.9)	0.059
Alcohol intake, n (%)	19 (10.0)	25 (15.3)	0.126	31 (15.2)	0.122	8 (8.7)	0.730
TG (mmol/L)	1.48 ± 0.72	1.75 ± 1.24	0.013*	1.63 ± 0.78	0.040*	1.39 ± 1.00	0.431
TC (mmol/L)	5.07 ± 1.02	5.20 ± 1.37	0.336	5.11 ± 1.17	0.725	4.88 ± 1.11	0.141
LDL-C (mmol/L)	3.46 ± 0.84	3.46 ± 1.00	0.974	3.44 ± 0.95	0.838	3.22 ± 0.87	0.028*
HDL-C (mmol/L)	1.42 ± 0.29	1.26 ± 0.34	<0.001*	1.28 ± 0.30*	<0.001*	1.22 ± 0.32	<0.001*
Ischemic heart disease, n (%)	11 (5.8)	14 (8.6)	0.307	6 (2.9)	0.164	13 (14.1)	0.019*
Atrial fibrillation, n (%)	4 (2.1)	7 (4.3)	0.238	2 (1.0)	0.617	67 (72.8)	<0.001*

SVO, small-vessel occlusion; CE, cardioembolism; TC, total cholesterol; TG, triglycerides; HDL-C, high-density lipoprotein cholesterol; LDL-C, low-density lipoprotein cholesterol; *p < 0.05.

### 3.2 P2Y12 Polymorphisms and Susceptibility to Different IS Subgroups

Table 2 presents the genotype distribution and allele frequencies of *P2Y12* polymorphisms in control participants and in patients with different stroke subtypes. Both SNPs conformed to the Hardy-Weinberg equilibrium in both the overall IS cohort and control group (all *p* > 0.05).

**Table 2.** **The genotype and allele frequencies of *i-T744C* and *C34T***.

SNP	Genotype/allele	Controls (n = 190)	LAA stroke (n = 163)	*p*	SVO stroke (n = 204)	*p*	CE stroke (n = 92)	*p*
*i-T744C*	Genotype							
	*TT*	146 (76.8)	100 (61.3)		150 (73.5)		63 (68.5)	
	*TC*	39 (20.5)	56 (34.4)		52 (25.5)		25 (27.2)	
	*CC*	5 (2.6)	7 (4.3)	0.007*	2 (1.0)	0.253	4 (4.3)	0.315
	Allele							
	*T*	331 (87.1)	256 (78.5)		352 (86.3)		151 (82.1)	
	*C*	49 (12.9)	70 (21.5)	0.002*	56 (13.7)	0.732	33 (17.9)	0.111
*C34T*	Genotype							
	*CC*	119 (62.6)	107 (65.6)		111 (54.4)		59 (64.1)	
	*CT*	64 (33.7)	48 (29.5)		74 (36.3)		28 (30.5)	
	*TT*	7 (3.7)	8 (4.9)	0.729	19 (9.3)	0.049*	5 (5.4)	0.716
	Allele							
	*C*	302 (79.5)	262 (80.4)		296 (72.5)		146 (79.3)	
	*T*	78 (20.5)	64 (19.6)	0.768	112 (27.5)	0.023*	38 (20.7)	0.972

SNP, single nucleotide polymorphism; *C34T*, a C→T transition at 34 nt downstream of exon 2’s start site of the *P2Y12* gene. *p < 0.05. TT, Thymine/Thymine homozygous genotype; CC, Cytosine/Cytosine homozygous genotype; TC, Thymine/Cytosine heterozygous genotype; T, Thymine; C, Cytosine.

The genotype distribution of the *i-T744C* polymorphism differed significantly between the LAA subtype and control groups, with the *C* allele being more frequent in LAA patients (21.5% vs. 12.9%, *p* = 0.002). Multivariate logistic regression analysis showed the *i-T744C* polymorphism was associated with a significantly increased risk of LAA stroke in both the dominant (adjusted odds ratio (OR) = 2.024, 95% confidence interval (CI): 1.191–3.440, *p* = 0.009) and additive (adjusted OR = 1.703, 95% CI: 1.078–2.692, *p* = 0.023) models following adjustment for relevant confounders (Table 3). A significant association between the *C34T* polymorphism and SVO subtype risk was observed in the recessive model via univariate logistic regression analysis (*p* = 0.025). However, this positive association was lost after adjustment for other covariates (adjusted OR = 1.854, 95% CI: 0.708–4.852, *p* = 0.209). Additionally, both univariate and multivariable logistic regression analyses found no statistically significant relationships between the *i-T744C* and *C34T* polymorphisms and CE subtype risk.

**Table 3.** **The different genetic model frequencies of *i-T744C* and *C34T***.

SNP		Genetic model		Crude OR (95% CI)	Crude *p*-value	Adjusted OR (95% CI)	Adjusted *p*-value
LAA stroke^a^							
	*i-T744C*	Dominant	*TT* vs. *TC+CC*	2.090 (1.318–3.316)	0.002*	2.024 (1.191–3.440)	0.009*
		Recessive	*TT+TC* vs. *CC*	1.660 (0.517–5.335)	0.390	1.165 (0.313–4.334)	0.820
		Additive	*TT* vs. *TC* vs. *CC*	1.818 (1.219–2.712)	0.003*	1.703 (1.078–2.692)	0.023*
	*C34T*	Dominant	*CC* vs. *CT+TT*	0.877 (0.567–1.358)	0.557	0.919 (0.557–1.516)	0.741
		Recessive	*CT+CC* vs. *TT*	1.349 (0.478–3.805)	0.570	1.356 (0.410–4.487)	0.618
		Additive	*CC* vs. *CT* vs. *TT*	0.946 (0.655–1.367)	0.769	0.978 (0.643–1.490)	0.919
SVO stroke^a^							
	*i-T744C*	Dominant	*TT* vs. *TC+CC*	1.195 (0.755–1.890)	0.447	1.408 (0.835–1.438)	0.199
		Recessive	*TT+TC* vs. *CC*	0.366 (0.070–1.911)	0.391	0.196 (0.032–1.205)	0.079
		Additive	*TT* vs. *TC* vs. *CC*	1.075 (0.712–1.622)	0.732	1.163 (0.729–1.441)	0.526
	*C34T*	Dominant	*CC* vs. *CT+TT*	1.404 (0.939–2.101)	0.098	1.372 (0.952–1.976)	0.090
		Recessive	*CT+CC* vs. *TT*	2.685 (1.102–6.540)	0.025*	1.854 (0.708–4.852)	0.209
		Additive	*TT* vs. *TC* vs. *CC*	1.185 (0.969–1.449)	0.099	1.429 (0.910–2.244)	0.121
CE stroke^b^							
	*i-T744C*	Dominant	*TT* vs. *TC+CC*	1.527 (0.878–2.658)	0.133	1.721 (0.711–4.162)	0.229
		Recessive	*TT+TC* vs. *CC*	1.682 (0.441–6.417)	0.684	2.476 (0.415–14.778)	0.320
		Additive	*TT* vs. *TC* vs. *CC*	1.432 (0.900–2.278)	0.129	1.625 (0.803–3.288)	0.176
	*C34T*	Dominant	*CC* vs. *CT+TT*	0.937 (0.559–1.573)	0.807	0.693 (0.285–1.689)	0.420
		Recessive	*CT+CC* vs. *TT*	1.502 (0.464–4.868)	0.495	1.202 (0.158–9.159)	0.859
		Additive	*TT* vs. *TC* vs. *CC*	1.008 (0.652–1.557)	0.972	0.786 (0.368–1.679)	0.535

^a^Adjusted for age, hypertension, diabetes mellitus, smoking, TG and HDL-C; ^b^Adjusted for age, gender, hypertension, ischemic heart disease, LDL-C, HDL-C, and atrial fibrillation; OR, odds ratio; *p < 0.05.

### 3.3 Screening of Variables and Establishment of the Nomogram in LAA Stroke

**Fig. 1.**
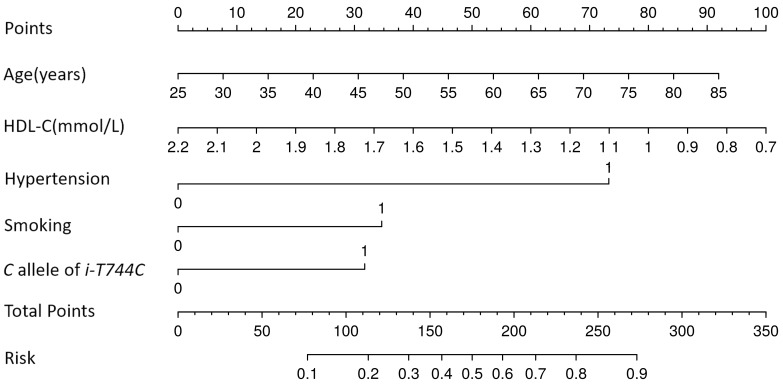
**Risk prediction nomogram for large-artery atherosclerosis stroke**. The predictors included age, hypertension, smoking, high-density lipoprotein cholesterol (HDL-C), and the *i-T744C* polymorphism. *i-T744C*, a T→C transition at 744 nucleotides (nt) downstream of intron 5’s start site of the *P2Y12* gene.

The characteristics of control and LAA patients in the training group are compared in Table 4. As shown in Table 5, univariate and multivariable logistic regression analyses identified five variables that appeared to be independent predictors of LAA stroke: age (OR = 1.033, 95% CI: 1.004–1.064, *p* = 0.026), hypertension (OR = 4.857, 95% CI: 2.804–8.577, *p* < 0.001), smoking status (OR = 2.207, 95% CI: 1.102–4.493, *p* = 0.027), HDL-C (OR = 0.264, 95% CI: 0.105–0.641, *p* = 0.004), and the *i-T744C* polymorphism (Thymine/Cytosine heterozygous genotype + Cytosine/Cytosine homozygous genotype (*TC+CC*) vs. Thymine/Thymine homozygous genotype (*TT*); OR = 2.021, 95% CI: 1.100–3.765, *p* = 0.024). Since all VIF values were <5, none of the variables were removed from the multivariable logistic regression. Consequently, a simple model for predicting LAA stroke was established by incorporating these independent risk factors into a nomogram (Fig. 1). IDI and NRI demonstrated that addition of the *i-T744C* polymorphism to the predictive model significantly improved the accuracy of LAA stroke risk prediction (IDI = 0.016, 95% CI: 0.002–0.030, *p* = 0.030; NRI = 0.278, 95% CI: 0.065–0.491, *p* = 0.011).

**Table 4.** **Variable characteristics of LAA stroke and controls in the training cohort**.

Variable	Controls (n = 152)	LAA stroke (n = 130)	*p*-value
Age (years)	59.40 ± 9.11	63.10 ± 10.10	0.001*
Men, n (%)	71 (46.7)	72 (55.4)	0.146
Hypertension, n (%)	48 (31.6)	95 (73.1)	<0.001*
Diabetes mellitus, n (%)	35 (23.0)	57 (43.8)	<0.001*
Cigarette smoking, n (%)	22 (14.5)	39 (30.0)	0.002*
Alcohol intake, n (%)	15 (9.9)	18 (13.9)	0.300
TG (mmol/L)	1.50 ± 0.74	1.74 ± 1.17	0.043*
TC (mmol/L)	5.09 ± 1.03	5.14 ± 1.37	0.739
LDL-C (mmol/L)	3.46 ± 0.84	3.42 ± 1.01	0.771
HDL-C (mmol/L)	1.42 ± 0.30	1.25 ± 0.34	<0.001*
Ischemic heart disease, n (%)	9 (5.9)	11 (8.5)	0.407
Atrial fibrillation, n (%)	4 (2.6)	5 (3.8)	0.563
*C* allele of *i-T744C*	35 (23.0)	48 (36.9)	0.011*

*p < 0.05.

**Table 5.** **Logistic regression analysis of LAA stroke-related risk factors in the training cohort**.

Variable	Univariate regression analysis		Multivariate regression analysis	
	OR (95% CI)	*p*-value	OR (95% CI)	*p*-value
Age	1.041 (1.015–1.068)	0.002*	1.033 (1.004–1.064)	0.026*
Hypertension	5.881 (3.507–9.861)	<0.001*	4.857 (2.804–8.577)	<0.001*
Diabetes mellitus	2.610 (1.564–4.357)	<0.001*	1.627 (0.899–2.951)	0.107
Cigarette smoking	2.532 (1.408–4.556)	0.002*	2.207 (1.102–4.493)	0.027*
TG	1.306 (1.010–1.689)	0.042*	1.054 (0.785–1.452)	0.734
HDL-C	0.171 (0.076–0.384)	<0.001*	0.264 (0.105–0.641)	0.004*
*C* allele of *i-T744C*	1.957 (1.164–3.288)	0.011*	2.021 (1.100–3.765)	0.024*

*p < 0.05.

**Fig. 2.**
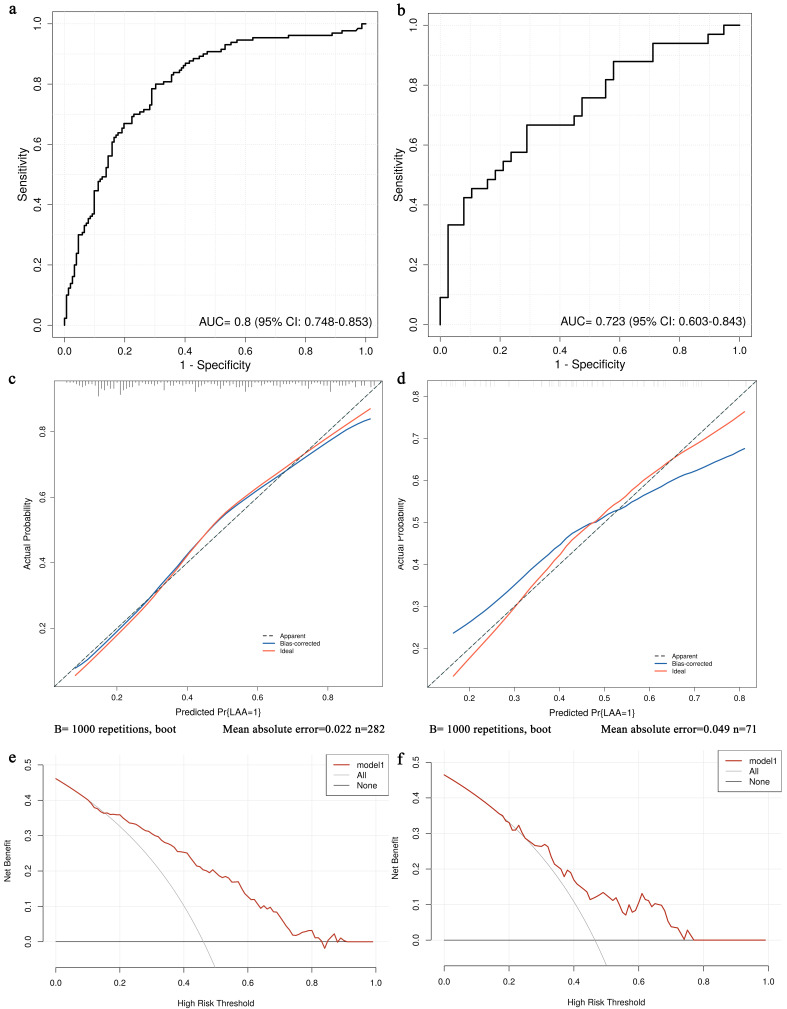
**Validation of the nomogram**. Receiver operating characteristic, calibration, and decision curve analysis of the training cohort (a,c,e) and validation cohort (b,d,f). ROC, receiver operating characteristic curve; AUC, area under the ROC curve; CI, confidence interval; LAA, large-artery atherosclerosis.

This predictive nomogram had AUCs of 0.8 (95% CI: 0.748–0.853) in the training cohort, and 0.723 (95% CI: 0.603–0.843) in the validation cohort, indicating consistent diagnostic efficacy for LAA stroke (Fig. 2a,b). The sensitivity and specificity were 0.8 and 0.697, respectively, in the training cohort, and 0.667 and 0.711, respectively, in the validation cohort. Furthermore, the calibration curve demonstrated good calibration of the predictive model (*p* = 0.392, Hosmer-Lemeshow test). The mean absolute error was 0.022 in the training group and 0.048 in the validation group (Fig. 2c,d). DCA revealed the model curves showed significant deviation from extreme values, thus demonstrating certain clinical utility in predicting LAA stroke (Fig. 2e,f). Collectively, these results indicate that the nomogram model incorporating the *P2Y12 i-T744C* polymorphism and clinical data holds substantial clinical significance for the prediction of LAA stroke risk.

## 4. Discussion

This study provides the first evidence of a significant association between the *P2Y12 i-T744C* polymorphism and an elevated risk of LAA stroke. Furthermore, we developed a nomogram model that incorporates traditional risk factors along with the *i-T744C* polymorphism for predicting LAA stroke. Patients with high scores in this predictive nomogram have a high probability of developing LAA stroke, and may therefore derive clinical benefit from early prediction and preventive care.

Studies have established that the P2Y12 receptor, encoded by the *P2Y12* gene, plays an essential role in atherosclerosis and atherothrombosis. Activation of P2Y12 on platelets triggers the phosphoinositide 3-kinase (PI3K)/protein kinase B (PKB/AKT) pathway via coupled G protein βγ (Gβγ) subunits, resulting in platelet activation and thrombosis [11, 19]. Moreover, activated P2Y12 facilitates the release of a variety of pro-inflammatory factors [8], thereby contributing to inflammatory cascades linked to atherosclerosis. Recent research has demonstrated that P2Y12 receptors participate in regulating VSMC migration into the intima and plaque, which contributes to the development of atherosclerosis and its complications [20].

Other investigations have shown that *P2Y12* genetic variants were associated with susceptibility to atherosclerosis-related disorders and also influenced platelet reactivity. Fontana *et al*. [10] reported that four variants in the *P2Y12* gene—*i-T744C*, *i-C139T* (a C→T transition at 139 nt downstream of intron 5’s start site), *i-ins801A* (a single-nucleotide A insertion at 801 nt downstream of intron 5’s start site), and *G52T* (a G→T transition at 52 nt downstream of exon 2’s start site)—were in linkage disequilibrium. An individual who carries all mutant alleles in the four polymorphisms was classified as an H2 haplotype carrier. Otherwise, they were classified as having the H1 haplotype. Most studies have used the *i-T744C* or *G52T* SNPs to represent the haplotypes H1 and H2 [13, 14, 15]. The *P2Y12* H2 haplotype was reported to be positively correlated with ADP-induced platelet aggregation and was accompanied by a significant reduction in the cyclic adenosine monophosphate (cAMP) concentration [21]. A case–control study reported that H2 allele carriers displayed significant susceptibility to peripheral arterial disease compared with H1 allele carriers [22]. Several reports in distinct populations have revealed that patients with mutant genotypes of the *i-T744C* polymorphism have a higher propensity for CAD or acute coronary syndrome [12, 23]. Notably, there is still controversy regarding the association between *P2Y12* polymorphisms and IS. Lu *et al*. [14] found that the H2 allele may confer an elevated risk of cerebral infarction. In support of this, a meta-analysis indicated the *P2Y12 i-T744C* polymorphism may be predictive of IS risk [24]. In contrast, a prospective cohort study conducted in white males yielded negative results [15]. Previous study has suggested that predisposition to certain IS subtypes is genetically determined, with distinct mechanisms underlying their development [17]. Consequently, the lack of specific IS subclassification and instead the investigation of overall IS association with *P2Y12* polymorphisms may lead to potential bias in studies of genotype-phenotype associations. Unlike other IS subtypes, the pathophysiological foundation of LAA stroke is the development of platelet-rich thrombus at the site of the ruptured atherosclerotic lesion [25]. Our analysis indicated the *i-T744C* polymorphism is an independent risk factor for the LAA subtype, but not for the SVO or CE subtypes. This finding supports the hypothesis that the *P2Y12 i-T744C* polymorphism exerts subtype-specific effects on LAA stroke, but not on other IS subtypes. The theoretical basis for this observation is grounded in the role of the *i-T744C* SNP in regulating platelet aggregation and mediating the development of atherosclerosis. However, future studies should also perform functional experiments to elucidate the molecular mechanisms underlying the *P2Y12 i-T744C*-LAA stroke genotype-phenotype association.

Consistent with previous findings [15], the *P2Y12 C34T* polymorphism did not correlate with any IS subtypes in the current study. The *P2Y12 C34T* polymorphism has also been investigated for its association with clopidogrel resistance (CR). The results showed the T allele of *C34T* polymorphism was associated with increased susceptibility to CR and concomitant adverse cardiac or cerebrovascular outcomes in Chinese cohorts [26]. However, conflicting results have been reported in other ethnic groups [27], and further studies should focus on correlations between the *C34T* polymorphism and adverse clinical outcomes in clopidogrel-treated stroke patients.

The usefulness of nomograms for the diagnosis and prognostic evaluation of IS and its subtypes has previously been reported through the integration of relevant risk factors [28, 29, 30]. For example, Chen *et al*. [28] devised a nomogram with good discrimination for the prediction of early IS. Their nomogram comprised 6 clinical parameters: gender, diabetes, family history, coronary heart disease, smoking, and age. Similar to a report in the literature [16], we identified age, hypertension, smoking, and HDL-C as four clinical correlates of LAA stroke. Additionally, we also found that the 744C polymorphism may be a potential predictor for the development of LAA stroke. IDI and NRI-common metrics quantify the incremental predictive value of one model over another in clinical research [31]. These suggested the *i-T744C* polymorphism might offer important additional clinical information for LAA stroke risk prediction. Based on the above findings, we constructed a novel nomogram for early LAA stroke prediction that includes the *i-T744C* polymorphism and clinical variables. This may serve as a reliable tool for personalized LAA stroke monitoring, as evidenced by its satisfactory calibration, discriminative capacity (AUC of 0.8 in the training cohort and 0.723 in the validation cohort), and favorable net clinical benefit. Our nomogram provides an immediate predicted risk of LAA stroke in clinical practice based on patient-specific variables, helping clinicians to rapidly identify high-risk individuals (predicted probability >30%). Once identified, these individuals may warrant closer clinical follow-up and timely intervention. Importantly, the visual nomogram offers a straightforward quantitative reference for shared decision-making by the clinician and patient, thereby improving adherence to preventive interventions in the susceptible population. We anticipate that this predictive model will be continuously upgraded and refined for future clinical applications by incorporating new variables and expanding the sample size to enhance its accuracy and practicality.

Our study had several limitations. First, the sample sizes of the stroke subtype and control groups were relatively small. Further studies with larger and ethnically diverse cohorts are needed to confirm our results. Second, complex gene-environment interactions drive the development of different stroke subtypes, whereas our predictive nomogram focuses on limited variables and is exclusively applicable to LAA stroke. Therefore, future studies should incorporate more genetic and environmental variations, as well as systematic analysis of gene-environment interaction effects within stroke subtypes. Finally, this analysis was limited to genotype-phenotype associations and requires further in-depth exploration of the potential mechanisms underlying the observed associations.

## 5. Conclusion

Our findings revealed that the *P2Y12 i-T744C* polymorphism was associated with LAA stroke, with the C allele being a significant predisposing factor. We constructed a nomogram model that combines the *i-T744C* polymorphism and clinical variables. This model displayed a favorable capacity to discriminate individual LAA risk, and could thus help to identify high-risk individuals and enable personalized prevention. Our findings require further validation in large-scale, multi-ethnic studies.

## Availability of Data and Materials

The datasets used and analyzed in the current article can be available from the corresponding author upon reasonable request.
